# Barriers in Accessing Postpartum Mental Health Care and Coping Strategies Among Migrant and Refugee Women With Postpartum Depression

**DOI:** 10.1002/puh2.70424

**Published:** 2026-10-14

**Authors:** Marjia Sultana, Ruheea Taskin Ruhee, Miraj Ahmed Bhuiyan, Towhid Hasan

**Affiliations:** ^1^ Institute of Nutrition and Food Science University of Dhaka Dhaka Bangladesh; ^2^ Japan Society for the Promotion of Science Tokyo Japan; ^3^ School of Economics Guangdong University of Finance & Economics Guangzhou China; ^4^ Department of Food Technology and Nutrition Science Noakhali Science and Technology University Noakhali Bangladesh

**Keywords:** coping strategies, help‐seeking behavior, migrant, postpartum depression, refugee

## Abstract

**Background:**

Postpartum depression (PPD) significantly impacts the well‐being of mothers and their families, with migrant and refugee women being particularly vulnerable due to unique stressors and limited access to care. Hence, this scoping review examines the specific barriers refugee and migrant women experiencing PPD face in accessing mental healthcare, as well as the diverse informal support strategies they rely on.

**Methods:**

This scoping review was conducted following the Preferred Reporting Items for Systematic reviews and Meta‐Analyses extension for Scoping Reviews (PRISMA‐ScR) guidelines. A comprehensive literature search was performed across multiple databases, including Scopus, Web of Science, PubMed, Ovid, Cochrane Library, Google Scholar, and ScienceDirect, to identify relevant studies published from database inception to October 30, 2025.

**Results:**

A total of 18 studies (out of 5453 references extracted from bibliographic databases) were included in this scoping review. Common barriers to help‐seeking included stigma, lack of culturally sensitive care, language difficulties, and family‐related barriers. The elements for seeking help were spiritual practices, support from family, friends, and peers, and community resources.

**Conclusion:**

This review highlights the need for culturally sensitive approaches to addressing PPD among migrant and refugee women. Understanding the barriers and informal support types they already use can inform the development of supportive policies and culturally appropriate mental health services to improve access to care and postpartum well‐being.

## Graphical abstract

Migrant and refugee mothers experiencing postpartum depression (PPD) encounter interconnected cultural, linguistic, healthcare, financial, family, and immigration‐related barriers to help‐seeking. Nevertheless, family and partner support, spirituality, community networks, culturally relevant support, physical activities, and personal resilience provide important pathways for coping and recovery. The findings emphasize the need for culturally responsive, accessible, and equitable peripartum mental health services that build upon existing informal support systems to improve maternal and child well‐being.

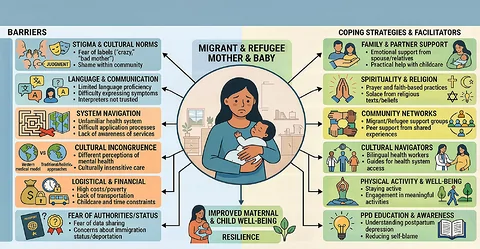

## 1 Introduction

The postpartum period is widely understood within the broader framework of the transition to parenthood, a major life‐course event involving substantial psychological, social, and role‐related adjustments [1, 2, 3]. During this transition, new parents must adapt to changes in identity, responsibilities, family dynamics, sleep patterns, and daily routines. Although this period can be joyful, it is often accompanied by increased emotional vulnerability and stress, making mothers more susceptible to mental health challenges, such as postpartum depression (PPD) [1, 2, 3]. According to the *International Classification of Diseases, 11th Revision (ICD‐11)*, PPD is classified under mental or behavioral disorders associated with the puerperium and typically occurs within the first weeks after childbirth, although symptoms may emerge anytime within the first postpartum year [4]. Although many parents experience short‐lived “baby blues,” PPD is more severe and can significantly impair daily functioning. Importantly, depressive symptoms may also begin during pregnancy and persist into the postpartum period [5].

The prevalence of PPD varies widely across countries. Hahn‐Holbrook et al. [6] estimated a pooled global prevalence of PPD of 17.7% across 56 countries. However, substantial variation in prevalence has been reported, with rates ranging from 3.9% to 69.9% across studies [7]. The burden of PPD is generally higher in developing countries, where prevalence during the first postpartum year has been reported to range from 15% to 57% [2]. This considerable variation and the disproportionately higher burden in developing settings may reflect differences in socioeconomic and social conditions, including poverty, limited social support, and restricted access to maternal mental health services. Common symptoms include helplessness, irritability, sleep disturbances, anxiety, hopelessness, loss of interest in usual activities, social withdrawal, and difficulties in bonding with the infant [7]. The etiology of PPD is multifactorial, involving genetic, physical, psychosocial, and obstetric factors. Trauma‐related experiences and attachment processes may also influence maternal mental health during the perinatal period [8]. If left untreated, PPD can negatively affect maternal well‐being, infant development, and family relationships and, in severe cases, may lead to self‐harm or harm to the infant [8].

Several treatment approaches are available for PPD, including psychotherapeutic and psychiatric care, parent–infant psychotherapy, family‐based interventions, and pharmacological treatment [5]. Perinatal care providers, such as midwives, also play a key role in screening, early identification, and referral to mental health services [5]. In addition to professional care, mothers may seek informal support from family members, friends, community networks, or religious leaders [9]. Such support may provide accessible assistance during the postpartum period and may serve as a pathway to professional care; however, it should complement rather than replace professional mental healthcare in cases of severe PPD. Access to specialized peripartum mental health services and professional treatment varies widely across countries and healthcare systems [2].

The global refugee and migrant population has reached approximately 25.9 million, with around 16% resettled in high‐income countries with cultural and social environments different from those of their countries of origin [9]. Refugee and migrant women may experience a higher burden of PPD because of greater psychological distress, social isolation, displacement‐related trauma, and the challenges of adapting to parenthood while simultaneously adjusting to a new country [5, 10, 11, 12, 13, 14]. For example, refugee, immigrant, and asylum‐seeking mothers in Canada experienced almost five times higher prevalence of PPD than Canadian‐born mothers [2]. These challenges are further compounded by difficulties accessing appropriate healthcare, as differences in language, culture, and healthcare systems may create barriers to timely and culturally appropriate assessment and treatment [9]. Thus, the simultaneous demands of adapting to parenthood and adjusting to a new social and cultural environment may intensify the risk of PPD while also limiting access to mental healthcare, potentially creating a cycle of increased need and reduced access to support [5, 12, 15].

Help‐seeking behavior in the context of PPD refers to the recognition of psychological distress and the pursuit of assistance to manage symptoms [11]. Among migrant and refugee mothers, help‐seeking behaviors are influenced by psychological, social, and structural factors, including cultural beliefs, economic constraints, limited access to healthcare, gender inequalities, racial discrimination, immigration status, and language barriers [16]. Although existing research has examined PPD among migrant and refugee mothers in specific regions [14, 17, 18, 19] or among particular ethnic groups [10, 11], a comprehensive synthesis of the barriers and coping strategies influencing their help‐seeking behavior across diverse contexts remains limited.

Given the unique mental health challenges faced by migrant and refugee mothers, this scoping review aims to examine their experiences of help‐seeking behavior in relation to PPD. By synthesizing evidence from studies conducted across different countries and cultural contexts, this review seeks to identify the key barriers and coping strategies shaping access to mental health support among migrant and refugee mothers. Understanding these factors is essential for informing culturally responsive policies and interventions that can improve maternal mental health outcomes and access to care during the postpartum period.

## 2 Methods

### 2.1 Literature Search Strategy

This scoping review adhered to the guidelines established by the Preferred Reporting Items for Systematic reviews and Meta‐Analyses extension for Scoping Reviews (PRISMA‐ScR) [20]. A literature search of the electronic databases, namely, Scopus, Web of Science, PubMed, Ovid, Cochrane Library, Google Scholar, and ScienceDirect, was carried out from their inception until October 30, 2025. The databases were searched using the following keywords: help seek^*^, refugee or migrant, postpartum or postnatal, and depression. The full search strategy is presented in Table 1. Only the research published in English was included. We aggregated the search results from the electronic sources utilizing the bibliographic management tool (EndNote, v21) and applied the method of Bramer et al. [21] to remove duplicate entries. The reference lists of included articles were also reviewed for additional relevant articles.

**TABLE 1:** Search strategy of the electronic databases.

Database	Search strategy
Scopus	(TITLE‐ABS‐KEY (“help seek*”) AND TITLE‐ABS‐KEY (refugee) OR TITLE‐ABS‐KEY (migrant) AND TITLE‐ABS‐KEY (postpartum) OR TITLE‐ABS‐KEY (postnatal) AND TITLE‐ABS‐KEY (depression))
Web of Science	(ALL = (“help seek*”) AND (ALL = (refugee) OR ALL = (migrant)) AND (ALL = (postpartum) OR ALL = (postnatal)) AND ALL = (depression))
PubMed	(“help seek*”[All Fields] AND (“refugee”[All Fields] OR “migrant”[All Fields]) AND (“postpartum”[All Fields] OR “postnatal”[All Fields]) AND “depression”[All Fields])
Ovid	(help seek* and (refugee or migrant) and (postpartum or postnatal) and depression).mp. [mp = title, abstract, full text, caption text]
Cochrane Library	(help seek*) in Title Abstract Keyword AND (refugee OR migrant) in Title Abstract Keyword AND (postpartum OR postnatal) in Title Abstract Keyword AND depression in Title Abstract Keyword
Google Scholar	((help seek*) and (refugee or migrant) and (postpartum or postnatal) and depression)
ScienceDirect	(“help seek” AND (refugee OR migrant) AND (postpartum OR postnatal) AND depression)

### 2.2 Study Selection

The eligibility criteria were established a priori, following the PICOS (Population, Intervention, Comparison, Outcome, Study design) framework. This review included studies involving migrant or refugee mothers diagnosed with PPD and focused on factors influencing help‐seeking behavior for PPD. No specific comparison group was required, and studies were included if they reported barriers and coping strategies to seeking mental healthcare. Different cross‐sectional, cohort, or case–control studies were considered, whereas systematic reviews, meta‐analyses, case reports, commentaries, and conference abstracts were excluded. Two independent reviewers screened the selected studies independently in two phases. Initially, titles and abstracts were screened to remove irrelevant studies. In the second phase, full‐text articles were assessed for eligibility. Any disagreements were resolved through discussion, with a third reviewer consulted when necessary. A PRISMA‐ScR flow diagram (Figure 1) illustrates the selection process.

**FIGURE 1:**
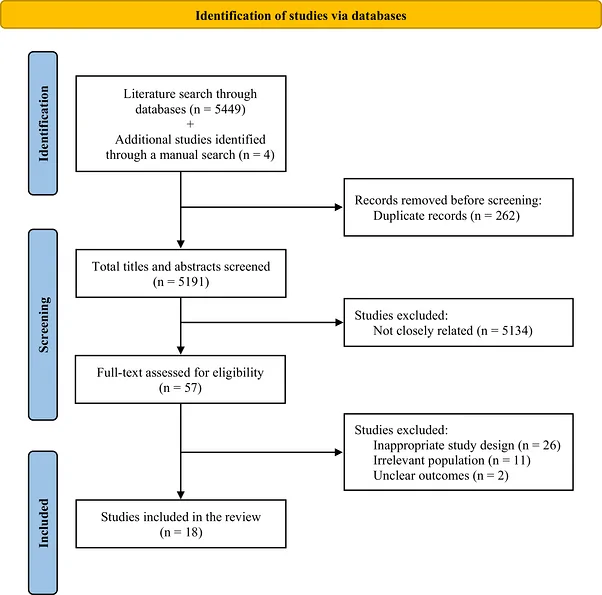
PRISMA flowchart.

### 2.3 Data Extraction

Data from the selected studies were independently extracted by two authors using a standard data extraction form, and discrepancies were resolved until agreement was reached. The following domains were extracted: publication year, author details, journal name, country, study design, participants (number and characteristics), and main findings. To ensure the maximum number of studies included in our review, we contacted the corresponding authors or co‐authors when the full‐text paper was unavailable.

### 2.4 Risk of Bias Assessment

In this review, the Newcastle–Ottawa Scale [22] was utilized to assess the risk of bias. It has been validated for use in observational studies and was widely used in numerous systematic reviews in psychiatry [23]. The scale evaluated each study based on eight items across three domains: the selection of the study groups, the comparability of the groups, and the ascertainment of the exposure or outcome. Each item was graded on a scale of 0–1 point, except for comparability, which allowed up to 2 points. The maximum achievable score was 9 points, with higher scores indicating higher quality. Studies scoring ≥7 were considered good quality, 4–6 moderate quality, and ≤3 low quality.

## 3 Results

### 3.1 Study Selection

Figure 1 shows a comprehensive flowchart of the literature screening process. The search strategy identified 5453 publications. After removing 262 duplicate records, 5191 papers were left to be screened based on their titles and abstracts. During this phase, 5134 studies were excluded as they were not closely related to the topic under investigation. In the eligibility assessment stage, 57 studies were examined in full to determine their suitability for inclusion. Of these, 39 studies were excluded for various reasons: 26 had inappropriate study designs, 11 focused on irrelevant populations, and 2 had unclear outcomes. Finally, 18 studies met all the criteria and were included in the review.

### 3.2 Study Characteristics

Eighteen studies conducted on refugee or immigrant mothers were included in the present scoping review (Table 2). All studies were published between 2008 and 2023, with samples from several countries, including Canada (8 out of 18), Australia (3 out of 18), the United States (3 out of 18), the United Kingdom (2 out of 18), Lebanon (1 out of 18), and 1 from multiple countries. The number of participants varied widely, ranging from 11 to over 18,000. All studies included mothers aged between 18 and 45 years. Participants were primarily first‐time mothers from diverse cultural backgrounds, including African, Asian Indian, Korean, Vietnamese, and Afghan origins.

**TABLE 2:** Summary of the characteristics and findings of the included studies.

				Summary of findings	
Study	Participant's number; characteristics (mothers experiencing postpartum depression)	Country of currently residing; country of origin	Study design; data collection tools	Barriers to asking for help	Coping strategies
Alnaji, Tippens [5]	8; refugees who were postpartum mothers	Informal camps in Lebanon; Syria	Qualitative phenomenological approach/mixed‐methods study; semi‐structured in‐depth Interview	Stigma of mental disease	Praying and reading Quran, crying, getting busy with kids
Baiden, Evans [16]	10; immigrant (new comer) mothers who birthed a baby in Canada within the past year	Southern Ontario, Canada; Africa	Qualitative study; open‐ended, semi‐structured interviews	Stigma surrounding mental illness in their home countries; temporary immigration status affected their eligibility for provincial medical insurance and subsequently access to postpartum mental health services; racial discrimination	Increasing newcomers’ awareness about maternal mental health and increasing availability of mental health services postpartum to facilitate utilization; getting information on postpartum mental health from health providers during pregnancy and after childbirth; stating preferences for helplines and online services; spiritual care and spousal support over seeking formal mental health services
Hamwi, Lorthe [8]	1475; postpartum migrant mothers	Brazil, Africa, Europe, Asia, America; Portugal	Prospective cohort study; computer‐assisted telephone interview	Language barriers	—
Rao, Dahlen [1]	11; migrant mothers gave birth in Canberra and were 6 weeks to 6 months postpartum	Canberra, Australia; India	Qualitative study; face‐to‐face semi‐structured in‐depth interviews	Stigma; supports from public healthcare services can be difficult due to several limitations such as less staffed, unavailability, inconsistent advices; limited understanding of Indian postpartum traditions among health providers, and culturally insensitive advice given by them	Getting supports from extended family members, husbands, and close friends; sharing feelings with other Indian neighbors who are in similar situations; support from midwives
Han, Goyal [25]	11; immigrant mothers aged >18 years who had given birth to a live infant within the past 12 months	The United States; Korean	Qualitative exploratory study; semi‐structured interviews	Cultural values (saving face) and stigma associated with mental illness (weak mindedness); frustration with language	Postpartum rituals; family supports; seek professional help only when the PPD symptoms increased
King, Feeley [26]	341; immigrant and Canadian‐born women with 12–14 weeks and 24–28 weeks of gestation as well as 1‐ and 8‐week postpartum	Canada	Longitudinal study; self‐reported measures	Stigma	—
Moore, Jayaweera [31]	10,333–10,838; migrant mothers within 9 months of childbirth	The United Kingdom; India, Pakistan, Bangladesh, Africa	Millennium Cohort Study; secondary analysis of data from the Millennium Cohort Study	Ethnic inequalities	—
Ta Park, Goyal [27]	15; mothers who had given birth to at least one live infant within the previous year	Northern California, the United States; Vietnam	Mixed‐methods pilot qualitative study; semi‐structured in‐depth interview	Stigma; lack of culturally appropriate care; lack of financial resources or health insurance shame/embarrassment; lack of knowledge about mental healthcare options; fear of the medication's side effects; denial and hopelessness	Seeking professional help if PPD conditions would be severe; significant role of family on help‐seeking; professional help‐seeking as last resort; preference for other methods; social support; complementary and alternative medicine; engage in other activities like exercising, shopping
Ahmed, Bowen [2]	12; refugees who were either pregnant or 1 year postpartum	Saskatoon, Canada migrated in 2015–16; Syria	Mixed‐methods research design; single focus group discussion and using a structured questionnaire	Stigma of mental health and privacy concerns; language difficulties that impacted their privacy especially when seeking healthcare services	Receiving social support around the time of birth, either during labor or afterward, with emphasis on emotional support; getting support from residing country with housing, medical care, and financial; spiritual practices, such as prayer and reading the Holy Quran as a source of support and strength; believing that support programs would be useful but to avoid stigma from their community programs should be named in a way that does not refer directly to depression but rather wellness; doing exercise like walking and swimming
Russo, Lewis [9]	38; migrant mothers having at least one Australian‐born child	Southeast region of Melbourne, Australia; Afghanistan	Qualitative study; focus group discussions and in‐depth interviews	Cultural stigma associated with mental illness; dissatisfaction with the maternity care they received within Australia; confliction with the cultural practices of Afghanistan	Support from partner; religion (performing associated prayer rituals); strong relationship with child; forming friendships, education, and utilizing childcare; self‐care and relaxation to improving the well‐being
Shafiei, Small [36]	39; immigrant mothers who were at 4 months of childbirth	Melbourne, Australia	Qualitative study; semi‐structured questionnaire among 39 participants further in‐depth face‐to‐face interview among 10 mothers	Believing in mind that health professionals could not help and may not want to deal with emotional issues; feel reluctant or uncomfortable to talk to a health professional especially male doctor; mother's idea or uncertainty about the role of health professionals; health professionals being rushed and not asking mothers about their mental health; minimizing mother's health issues by caregivers	—
Goyal [28]	12; immigrant mothers giving a childbirth within the last 12 months	India; Northern California	Qualitative study; semi‐structured interviews	Stigma as a barrier to obtaining mental healthcare; wanting to seek mental healthcare anonymously; feelings of personal failure for needing mental healthcare; seeking professional help as a last resort; help‐seeking influenced by generational values	Importance of mental healthcare providers understanding the culture; preference of social support and complementary/alternative therapies
Gardner, Bunton [29]	6; migrant mothers experiencing low mood in the postnatal period	Manchester, the United Kingdom, West Africa	Qualitative study; semi‐structured interviews	Fear of being stigmatized	Spiritual and religious faith; relationship with self, baby, friends, and family
Mamisachvili, Ardiles [30]	17; immigrant mothers within 1 year postpartum	Canada; Chile, Peru, Uruguay, Guatemala. Portugal, Italy, Greece, Poland, Estonia, Croatia, Ethiopia, Rwanda, Hong Kong, India, Australia	Exploratory qualitative study; semi‐structured interviews	Fear of mental health stigma; parents’ lack of understanding of postpartum mood problems	—
O'Mahony, Donnelly [32]	30; 8 were refugee mothers and 22 immigrant mothers	Canada; Mexico, Brazil, Columbia, Costa Rica, Philippines, India, Pakistan, China, Middle East, Africa	Qualitative study with critical ethnography method; semi‐structured in‐depth interview	Complex gender‐related problems; poverty; insecure immigration status coupled with emotional and economic dependence; discrimination and poor spousal relationships and support; being unable to work; limited access to healthcare services; no access to language classes and/or housing; sadness and worry due to leaving family behind; shifting roles within the family from employed mother to stay‐at home mother	—
O'Mahony, Donnelly [34]	30; immigrant and refugee mothers	Canada	Critical ethnography method; in‐depth critical ethnographic interviews	Lack of language skills; limited resources/poverty; environment changes; employment transitions; social inequity; limited access to information about their rights; fear of social isolation and fear of losing their infant and/or being deported; unsettled immigration status; dominance and control of partners	Having instinctive survival and adaptation strengths; spiritual and religious practices as sources of strength and hope; having great optimism and willingness to move forward into the future
O'Mahony, Donnelly [35]	30; 8 were refugee mothers and 22 immigrant mothers	Western province of Canada; Central and South America, China, Middle Eastern countries, and South Asia	In‐depth interviews; semi‐structured interview	Difficulties in accessing healthcare services; lack of information about PPD; poor relationships with healthcare providers; tendency of healthcare providers to normalize the depressive feelings and downgrading the seriousness of their PPD conditions; language difficulties; lack of familiarity with healthcare services or unawareness of the existence of healthcare services	Formal support and services provided by healthcare providers and other community resource agencies; informal support given by partners, family members, and friends
Ahmed, Stewart [33]	10; refugee, asylum‐seeking, non‐refugee, and immigrant new mothers with 12 months following the birth	Toronto, Canada; 2 from China, 2 from India, 1 from Pakistan, 3 from South America, 1 from Egypt, and 1 from Haiti	Qualitative study; semi‐structured interviews	Language difficulties; embarrassment, stigmatization, or fear of being found an unfit mother; attitude of the people staffing the service centers; discouraged by staff who did not seem genuinely interested in helping them	Informal social support from families and friends; informal support from community; sharing experiments and feelings with other mothers experiencing the same; going through cognitive and psychological adjustment or modifying daily routines to cope with new situation

Among the included studies, 12 employed qualitative methods; 11 collected data through semi‐structured, in‐depth interviews, and 1 conducted a focus group discussion followed by in‐depth interviews. Three studies used mixed‐method approaches, two of which conducted semi‐structured interviews, and one applied a single focus group discussion with a structured questionnaire. The remaining three quantitative studies collected information using computer‐assisted telephone interviews, self‐reported measures, or secondary data analysis. Several diagnostic tools were used to assess PPD, namely, the Edinburgh Postnatal Depression Scale, Postpartum Depression Screening Scale, Kessler Psychological Distress Scale (K10), and various types of interviews, including semi‐structured and in‐depth interviews. The prevalence of PPD varied significantly across studies, ranging from 11% to 41%. Table 3 represents the risk of bias assessment for the included studies. All studies were considered high‐quality, as evidenced by a score of 5 or higher on the Newcastle–Ottawa Scale.

**TABLE 3:** Risk of bias assessment of included studies using Newcastle–Ottawa Scale.

	Selection				Comparability	Outcome			
Study	Representation of exposed cohort	Selection of the nonexposed cohort	Ascertainment of exposure	Outcome not present at the start of study	Comparability of cohorts on the basis of study design/analysis	Assessment of outcome	Follow‐up long enough for outcomes to occur	Adequacy of follow‐up of cohorts	Total
Alnaji, Tippens [5]	★	—	★	★	★	★	—	—	5★
Baiden, Evans [16]	★	—	★	★	★	★	—	—	5★
Hamwi, Lorthe [8]	★	★	★	★	★★	★	—	—	7★
Rao, Dahlen [1]	★	—	★	★	★	★	—	—	5★
Han, Goyal [25]	★	—	★	★	★	★	—	—	5★
King, Feeley [26]	★	—	★	★	★	★	★	★	7★
Moore, Jayaweera [31]	★	★	★	★	★★	★	★	★	9★
Ta Park, Goyal [27]	★	—	★	★	★	★	—	—	5★
Ahmed, Bowen [2]	★	—	★	★	★	★	—	—	5★
Russo, Lewis [9]	★	—	★	★	★	★	—	—	5★
Shafiei, Small [36]	★	—	★	★	★	★	—	—	5★
Goyal [28]	★	—	★	★	★	★	—	—	5★
Gardner, Bunton [29]	★	—	★	★	★	★	—	—	5★
Mamisachvili, Ardiles [30]	★	—	★	★	★	★	—	—	5★
O'Mahony, Donnelly [32]	★	—	★	★	★	★	—	—	5★
O'Mahony, Donnelly [34]	★	—	★	★	★	★	—	—	5★
O'Mahony, Donnelly [35]	★	—	★	★	★	★	—	—	5★
Ahmed, Stewart [33]	★	—	★	★	★	★	—	—	5★

### 3.3 Study Theme

The findings were organized into two overarching themes: (1) barriers to help‐seeking for PPD, including stigma, cultural and language barriers, financial constraints, family‐related challenges, and limited healthcare access; and (2) coping strategies and sources of support, including family and community support, spiritual practices, self‐adaptation, and professional care (Table 2).

#### 3.3.1 Barriers to Help‐Seeking Behavior

##### 3.3.1.1 Fear of Stigma

Stigma arises when people endorse prejudice and discrimination against a person with mental health difficulties and problems. It becomes a substantial and multifaceted barrier to accessing healthcare for mental health difficulties [24]. In this review, fear of stigma was one of the most frequently reported barriers to help‐seeking and was identified in 12 of the 18 included studies [1, 2, 5, 9, 16, 25, 26, 27, 28, 29, 30]. Many migrant and refugee mothers expressed concern about being judged or stigmatized by their communities if they disclosed symptoms of PPD. In some cultural contexts, mothers feared being perceived as weak or incapable of fulfilling maternal responsibilities [5, 16]. As a result, they tried to avoid disclosing their problems with their friends or community for fear that their depression would be revealed [30].

##### 3.3.1.2 Problems Arise Due to Staying in Country Other Than Home Country

Limited access to healthcare and mental health services due to immigration or refugee status was reported in several studies (approximately 10 studies) [1, 2, 8, 17, 24, 26, 33, 34, 35]. Adapting to a new country, either as a refugee or an immigrant, is difficult for a new mother. Cultural dissimilarities [25], lack of culturally appropriate care services [1, 9, 27], limited employment opportunities, limited access to healthcare services, gender inequities, racial disparities [16, 31], unpredictable immigration status [16, 32], fear of social isolation [33, 34], and language differences [2, 8, 25, 32, 33, 34, 35] are associated with staying in country other than their native countries. All of the above factors were reported as barriers in seeking mental healthcare services by the refugee or immigrant mothers. In Canada, temporary immigration status sometimes affected eligibility for provincial health insurance, thereby limiting access to postpartum mental health services [16].

##### 3.3.1.3 Family‐Related Barriers

Family‐related barriers were identified in five studies [17, 29, 31, 33, 35]. The participants of a few studies faced a conflict with their families, especially with their parents or in‐laws, regarding their parenting methods. In addition, the parents did not want to understand the postpartum mood problems and remained more unsupportive [30]. Sometimes, older family members perceived PPD as a sign of maternal weakness and interpreted mothers’ expressions of depressive symptoms as attempts to gain attention [28]. However, these discordances may be attributed to the cultural and age or generational effects [30]. In a few studies, mothers reported unsupportive spousal relationships or domestic violence [32]. Hence, they could not share their postpartum depressive conditions with their partners and family members. Besides, their dependence on family members or spouses for immigration and economic support made it difficult for them to leave these relationships, leading them to cope with PPD silently rather than seek mental healthcare services [34].

Moreover, in the case of a few immigrant/refugee mothers, their roles in the family suddenly changed because of moving to a new country and having a baby. The women who had worked outside and earned money earlier in their home country now had to stay home with the baby because of limited employment opportunities in the country they migrated to. Consequently, they felt valueless in their family, tried to hide their PPD, and did not seek any mental health services [16].

##### 3.3.1.4 Barriers Related to Healthcare Services

Barriers related to healthcare services were reported in nine studies [1, 24, 26, 28, 29, 33, 34, 35, 36]. In the studies, a few mothers reported negative feelings toward healthcare providers. They did not feel confident that health professionals could adequately address their emotional concerns, leading them to be reluctant to disclose their depressive symptoms to healthcare providers [36]. Moreover, the lack of culturally adapted care left mothers confused and unsure about whose advice to follow (older members of the family or health professionals of the current country) [1].

Few participants reported that, as refugees, they had limited access to healthcare services [32]. They did not have sufficient information about PPD and had poor connections with the care providers [34]. Even some people were unaware of the existence of healthcare services [27, 36], and participants who had knowledge of it considered seeking professional help as last resort or only when PPD increased [25, 28]. The majority of the mothers noticed that doctors do not provide sufficient time and do not want to listen to them carefully. The majority of mothers reported that doctors did not provide sufficient time or listen to them attentively. Healthcare providers were often perceived as being rushed and reluctant to discuss possible mental distress during antenatal and postnatal checkups [33]. In some cases, healthcare providers minimized mothers’ depressive symptoms by downplaying the severity of their condition, which ultimately discouraged them from seeking help for PPD [34, 35]. Limited financial resources and lack of health insurance were also reported as barriers to accessing postpartum mental health service [27, 32, 34].

##### 3.3.1.5 Barriers Related to Personal Issues

Personal barriers were identified in five studies [2, 28, 33, 35, 36]. Few mothers felt shy or embarrassed to express their feelings to others, particularly to male doctors, and some were hopeless, considering depression as a personal failure [36]. They were also concerned about the potential side effects of medications [27]. In some cases, mothers preferred to consult with health professionals anonymously [2]. Besides, most of the immigrant/refugee mothers, especially the Muslim ones, did not engage in activities, such as exercising, walking, swimming, shopping, and other indoor and outdoor activities, to help manage their PPD symptoms as they considered it a part of Western culture [2, 27].

##### 3.3.1.6 Barriers Related to Culture and Language

Cultural and language barriers were reported in four studies [2, 8, 24, 26]. For migrant mothers, limited proficiency in the local language may make adaptation to a new country more difficult by restricting communication and participation in social and cultural activities. Language difficulties may also contribute to social isolation and hinder mothers’ adjustment to their new environment [2]. In addition, difficulties understanding or expressing emotions to healthcare providers may cause communication anxiety and emotional distress during healthcare encounters. Poor language proficiency has been associated with higher levels of postpartum depressive symptoms among migrant women [8]. For example, a cohort study of migrant women who gave birth in a Portuguese public hospital found that poor language proficiency was associated with a higher prevalence of PPD [8]. Language barriers may also limit mothers’ ability to understand healthcare information, communicate their emotional concerns, and navigate healthcare services [25], thereby discouraging follow‐up and reducing access to appropriate postpartum mental healthcare [35]. Thus, language difficulties may simultaneously hinder adaptation to the host country, exacerbate psychological distress and PPD, and create barriers to accessing and continuing appropriate healthcare.

#### 3.3.2 Coping Strategies or Other Support Routes to Help‐Seeking Behavior

The majority of the mothers suffering from PPD tended to minimize their PPD symptoms either by normalizing PPD or trying to endure without help. They believed that PPD is normal and everyone has to go through it [25]. However, despite the difficulties associated with adapting to a new country, mothers tried to manage their PPD through the following strategies during or after childbirth (Table 2).

##### 3.3.2.1 Spiritual or Religious Practices

Spiritual or religious coping strategies were reported in five studies [2, 5, 9, 30, 37]. Spiritual or religious practice was one of the prominent coping mechanisms of PPD, followed by most of the participants. Doing prayer or reading the Holy Quran was mentioned as a significant alternative to managing their mental health problems in the postpartum period [2]. Some mothers reported that reading religious texts helped them cope with emotional distress and provided a sense of comfort during the postpartum period [29]. Connection to religion and performing related religious rituals help manage mothers’ emotional state [9]. Besides, practicing specific religious traditions can be a minor group; in that case, collaborative work, group discussion about religious rituals, and positive thinking from similar fellows may support overcoming the depression or anxiety of the mothers uniquely [5]. In a prospective longitudinal study of 106 mostly low‐socioeconomic‐status Appalachian pregnant women, Clements et al. [37] found that higher levels of social support were associated with fewer depressive symptoms across the study period, whereas greater religious commitment was inversely associated with depressive symptoms at 6 months postpartum.

##### 3.3.2.2 Getting Support From Family, Friends, and People From Own Country

Informal social support from family members, partners, or community members was one of the most commonly reported coping strategies and appeared in multiple studies (approximately eight studies) [1, 9, 17, 24, 26, 28, 30, 34]. Most of the mothers preferred to seek help from the family first, specially from their spouse, rather than seeking professional help [25]. Attachment or bonding with baby was also a possible alternative for the mothers to improve their mood [9]. Some mothers reported feeling supported through the developing attachment relationship with their infants [29]. Many mothers reported that their family members helped them by cooking, caring for newborns, or handling other household chores. Some participants appreciated their husbands’ efforts to relieve their sadness, either by talking with them or by taking care of the newborn. Support from friends helped mothers feel less isolated, which might be especially relevant in the context of migration [33].

##### 3.3.2.3 Seeking Professional Help Only When the PPD Symptoms Increased

Participants of the five studies preferred to consult with the health professionals only when the symptoms of PPD increased [17, 24, 26, 28, 29]. They considered mental healthcare a last option [25, 27].

##### 3.3.2.4 Engage in Extracurricular Activities

A few participants engaged in activities, such as exercising, walking, swimming, shopping, and other indoor and outdoor activities, to help manage their PPD symptoms. Meditation, such as yoga, was another way of adaptation to PPD reported in three studies [2, 28].

##### 3.3.2.5 Getting Help From the Current Country of Residence

Support from the host country was mentioned in two studies only [2, 24]. In particular, mothers appreciated the medical, housing, and financial assistance available in their country of residence, which they perceived as helping them cope with postpartum depressive symptoms [2].

##### 3.3.2.6 Having Instinctive Survival and Adaptation Strengths

Some participants described adapting to their new circumstances by adjusting their daily routines and developing new ways of managing their responsibilities and emotional difficulties [34, 35]. In addition, some mothers reported relying on their own motivation to identify and address their problems independently rather than immediately seeking external support [33].

## 4 Discussion

This scoping review synthesized evidence on the barriers and coping strategies influencing help‐seeking behavior for PPD among migrant and refugee mothers. The findings indicate that help‐seeking is shaped by a complex interaction of individual, cultural, and structural factors. The most frequently reported barriers included stigma, cultural and ethnic disparities, language difficulties, racism, spiritual beliefs, and financial constraints. In contrast, fewer studies highlighted coping strategies that supported recovery and maternal well‐being, such as family support, spiritual practices, and community resources.

### 4.1 Barriers to Help‐Seeking Behavior

Barriers related to help‐seeking behavior can be categorized into individual, cultural, and structural levels. Individual‐level barriers are multifaceted and include psychological and emotional factors, like stigma [5], emotional vulnerability [38], and reluctance or discomfort to express feelings to healthcare providers [36]. Additionally, the lack of knowledge about PPD and available resources [39] or low socioeconomic circumstances [40] or insufficient household income [13] further discourages help‐seeking behavior. Additionally, family conflicts, especially with parents or in‐laws, and unsupportive or abusive relationships prevent many mothers—particularly immigrants and refugees—from sharing their struggles or accessing mental health services. Dependence on spouses or family members for economic and immigration‐related support, feelings of devaluation following changes in family roles, financial constraints, lack of insurance, fear of stigma, concerns about medication side effects, and preferences for anonymous consultations further complicate mothers’ ability to seek help.

Among the cultural‐level, language barriers are persistent and can often be the cause of living an isolated immigrant life [8, 41]. Inability to speak the local language can create communication barriers for migrant mothers and has been associated with an increased risk of PPD [8]. Language difficulties may contribute to emotional distress, particularly when mothers are unable to express themselves in their mother tongue, and may result in dissatisfaction with care, difficulty expressing emotions, and feelings of being misunderstood [42]. However, some of the immigrant mothers benefited from the healthcare personnel despite time limitations and language difficulties [33].

Stigma surrounding mental health problems was identified as an important barrier to help‐seeking among migrant and refugee mothers. In some cultural contexts, symptoms of PPD may be perceived by family members as a normal emotional response to childbirth rather than as a mental health condition requiring professional care. Such perceptions may lead mothers and their families to normalize or conceal depressive symptoms because of concerns about shame, social judgment, or discrimination. In some cases, mothers relied on religious practices to cope with their emotional distress and to manage concerns related to stigma, rather than seeking professional mental healthcare [7]. Stigma generally refers to negative societal attitudes, discrimination, or feelings of shame associated with mental health conditions and can discourage mothers from disclosing symptoms or seeking appropriate care [30]. In the present review, 12 studies identified stigma as a barrier to maternal mental healthcare after childbirth (Table 2). For example, fear of stigma was reported as a major concern among West African mothers living in the United Kingdom, which influenced their willingness to seek mental health support [29].

Additionally, limited access to healthcare services is directly or indirectly linked to language difficulties and stigma. Mothers expected healthcare support to be related to their traditional practices, which conflicted with the support from the country's healthcare facilities [1]. It is widespread in Asian mothers to prioritize physical health over mental health because seeking help for mental health concerns is similar to attention‐seeking behavior [28]. Interestingly, around 60% of the participants did not need any help from healthcare professionals [43]. The precarious status of migrants, poverty, gender discrimination, and low‐income family support can directly affect mothers’ access to healthcare facilities. It is essential to support migrant mothers with culturally appropriate care, along with providing bilingual healthcare providers, so that migrant mothers can choose to receive healthcare facilities to overcome PPD [30, 32].

Lack of access to healthcare can be considered a primary structural‐level barrier to migrant women's help‐seeking behavior. It creates a sense of insecurity that, coupled with emotional and economic dependency, makes the mothers more vulnerable; therefore, it becomes difficult to recover from PPD [40]. Besides, immigration laws and policies limit access to specific healthcare services, including postpartum mental health services, making the mother emotionally fragile [32]. However, migrants holding permanent immigration status may get the privilege of health coverage, whereas newcomer migrant mothers may not be able to access mental health services due to high costs. In addition to that, getting a well‐paid job is troublesome because of the immigration status and racial discrimination, as the professional credentials of the migrants are not properly acknowledged on the same yardstick as those in the home country; either they get paid jobs or remain unemployed, thus leaving them with frustration and anxiety. Losing a job right after the birth of the baby, financial crisis, failing to send the baby to a care center due to high cost, accessing limited job opportunities while compromising professional expertise, and so forth cause the outset of hard times and misery that continue to depression [44].

### 4.2 Coping Strategies to Help‐Seeking Behavior

Despite facing multiple barriers, some mothers were still able to access support and recover from PPD due to certain coping strategies. Notably, these coping strategies were not simply the inverse of the barriers. Rather, individual life circumstances, such as personal resilience, adaptability, and intrinsic motivation, played a unique role. The ability to adjust to a new cultural environment or integrate medication into daily routines required exceptional self‐discipline and proactive coping strategies [33, 38, 44, 45].

Religious practices are among the coping mechanisms used in times of stress. Five studies from this review reported that receiving help through spiritual and religious faith [29], prayers and Quran recitals [2, 5], and religious rituals [9]. Literature suggested that religion might have direct/indirect positive/adverse effects on coping with mental health issues [46, 47]. Besides, some mothers preferred listening to group counseling rather than using drugs as a treatment for PPD due to religious reasons [48, 49]. Such findings represent that religious practices act as a protector from mental health problems.

Support from family or neighbors in similar situations is considered one of the key factors in recovering from PPD [45]. Sharing emotions with someone who is in a similar condition helps to relieve the mother's anxiety to some extent. Besides, living as a part of an extended family in a different country other than the home country also facilitates the improvement of mothers’ stress [1, 25]. Keeping some time away from the children has a positive influence on mothers’ emotional well‐being, whereas other family members can do the childcare [9]. A study conducted with Vietnamese immigrant mothers living in the United States reported that family‐oriented interventions potentially play an important role in both the development and recovery of PPD [27]. Emotional and practical support from a male partner was uniquely protective and irreplaceable. Mothers who lacked partners’ support were significantly more likely to experience PPD, even if they had support from friends and family [50]. Besides, participating in cultural gatherings and sharing thoughts with friends further helped to normalize the depressed mood [48]. Mothers from different cultural or linguistic backgrounds were able to access culturally aligned support by participating in internet forums and virtual parenting groups, which often offered emotional relief and practical advice tailored to their needs [51].

Migrant mothers often possess instinctive survival and adaptation strengths developed through experiences of migration, adversity, and caregiving. These inner resources—such as resilience, determination, and strong cultural or spiritual values—act as vital coping mechanisms during the postpartum period. A strong willingness to move forward further supports recovery, as mothers actively seek ways to improve their circumstances. Building knowledge, setting goals, and developing problem‐solving skills help foster self‐efficacy and emotional healing. Moreover, engaging in physical and leisure activities, like swimming, yoga, shopping, or exercising, also facilitates alleviating depressive symptoms [2, 27, 52]. Besides, complementary and alternative medicine is also preferred to address depression [27]. These activities helped manage emotional distress and served as empowering routines that fostered a sense of control in an unfamiliar environment. Among the complementary therapies, yoga is a safe and acceptable intervention, showing beneficial effects in the management of depression and anxiety [53].

Professional mental healthcare is often viewed as a last option, pursued only after other personal or cultural coping strategies have been exhausted. In some cases, the willingness to seek help increases when individuals reside in countries where mental health services are more available and socially accepted [27].

Our findings indicate that access to support and treatment for PPD among migrant women is influenced by intersecting factors, including stigma, racism, limited language proficiency, restrictive immigration status, spiritual conflicts, ethnic inequalities, and financial instability. These barriers were further compounded by a lack of trust in health services and limited access to culturally appropriate care, leading some women to seek support from informal sources rather than the formal healthcare system. However, several coping strategies were found to positively influence help‐seeking behavior and psychological recovery. Although these coping strategies do not simply mirror the barriers in reverse, they highlight the importance of personal resilience and adaptive coping in the face of systemic adversity. Therefore, future research and intervention strategies should extend beyond addressing structural and cultural barriers encountered by migrant mothers to also enhancing the supportive resources and social networks that foster their empowerment. Key measures include the expansion of culturally competent services, ensuring equitable access irrespective of immigration status, and fostering active family and community involvement—each of which is essential for mitigating the prevalence and impact of PPD among migrant women.

## 5 Strength and Limitations

The 18 studies included in our scoping review offered several strengths. The primary strength was that the review provided a comprehensive analysis of postpartum mental health among immigrant and refugee mothers, covering a diverse population and multiple settings. Hence, it contributed to a broad understanding and offered a global perspective on this issue. Additionally, the use of the Newcastle–Ottawa Scale to assess the risk of bias added rigor to the evaluation of included studies and enhanced the clarity of the findings. Furthermore, the review identified a critical gap in maternal healthcare by highlighting the factors contributing to help‐seeking behavior, which could direct future interventions and policies to ameliorate maternal mental health services for these populations.

This scoping review had several limitations. First, the included studies exhibited considerable heterogeneity, encompassing diverse populations from different cultural backgrounds and settings with distinct healthcare systems, which posed challenges to the generalizability of the findings. Second, most of the included studies had relatively small sample sizes, which may have limited the robustness and representativeness of their findings. Third, language barriers and translation issues, along with cultural differences highlighted in the studies, might have introduced bias and influenced the accuracy of the findings. Finally, methodological differences, including varied study designs and assessment tools, might have complicated direct comparisons and synthesis of the results.

## 6 Conclusion

It is evident from the present scoping review that PPD is a serious concern among immigrant and refugee mothers. The barriers attributed to seeking help commonly include stigma, lack of culturally sensitive care, and language difficulties. Despite these challenges, many mothers find support through spiritual practices, from family, friends, and peers, and community resources. This review highlights the resilience of these mothers and underscores the significance of social support systems in their recovery process. It also advocates for policy implementations, including increased funding, better access to care, and legal protections for migrant and refugee mothers. Overall, the findings prioritize the need for a multifaceted approach involving clinical practice, policy modification, community support, and targeted research to efficiently address and mitigate PPD among migrant and refugee mothers, thereby uplifting their mental health and well‐being.

## Author Contributions

**Marjia Sultana**: conceptualization, data curation, visualization, writing – original draft. **Ruheea Taskin Ruhee**: data curation, visualization, writing – original draft. **Miraj Ahmed Bhuiyan**: conceptualization, formal analysis, validation, writing – review and editing. **Towhid Hasan**: conceptualization, writing – review and editing, formal analysis, validation.

## Funding

The authors have nothing to report.

## Ethics Statement

The authors have nothing to report.

## Consent

The authors have nothing to report.

## Conflicts of Interest

The authors declare no conflicts of interest.

## Data Availability

Not applicable because no new data or databases were used in the preparation of this work.
